# Genetic identity and differential gene expression between *Trichomonas vaginalis *and *Trichomonas tenax*

**DOI:** 10.1186/1471-2180-9-58

**Published:** 2009-03-18

**Authors:** Ashwini S Kucknoor, Vasanthakrishna Mundodi, JF Alderete

**Affiliations:** 1 School of Molecular Biosciences, Washington State University, Pullman, WA, USA

## Abstract

**Background:**

*Trichomonas vaginalis *is a human urogenital pathogen responsible for trichomonosis, the number-one, non-viral sexually transmitted disease (STD) worldwide, while *T. tenax *is a commensal of the human oral cavity, found particularly in patients with poor oral hygiene and advanced periodontal disease. The extent of genetic identity between *T. vaginalis *and its oral commensal counterpart is unknown.

**Results:**

Genes that were differentially expressed in *T. vaginalis *were identified by screening three independent subtraction cDNA libraries enriched for *T. vaginalis *genes. The same thirty randomly selected cDNA clones encoding for proteins with specific functions associated with colonization were identified from each of the subtraction cDNA libraries. In addition, a *T. vaginalis *cDNA expression library was screened with patient sera that was first pre-adsorbed with an extract of *T. tenax *antigens, and seven specific cDNA clones were identified from this cDNA library. Interestingly, some of the clones identified by the subtraction cDNA screening were also obtained from the cDNA expression library with the pre-adsorbed sera. Moreover and noteworthy, clones identified by both the procedures were found to be up-regulated in expression in *T. vaginalis *upon contact with vaginal epithelial cells, suggesting a role for these gene products in host colonization. Semi-quantitative RT-PCR analysis of select clones showed that the genes were not unique to *T. vaginalis *and that these genes were also present in *T. tenax*, albeit at very low levels of expression.

**Conclusion:**

These results suggest that *T. vaginalis *and *T. tenax *have remarkable genetic identity and that *T. vaginalis *has higher levels of gene expression when compared to that of *T. tenax*. The data may suggest that *T. tenax *could be a variant of *T. vaginalis*.

## Background

Trichomonads constitute a group of protists belonging to the phylum Parabasala that are mostly parasitic or commensal flagellates inhabiting oxygen-poor environments [1]. *Trichomonas vaginalis *is responsible for the number one, non-viral sexually transmitted disease (STD) with ~9 million new cases of women with trichomonosis in the US alone, and 250–350 million worldwide [2-5]. This STD causes serious adverse health outcomes in women, including adverse pregnancy outcomes, cervical neoplasia, atypical pelvic inflammatory disease, and infertility [6-8]. Men with trichomonosis may have non-gonococcal urethritis, prostatitis, epydidymitis, and infertility [8,9]. A relationship has been established between trichomonosis and cervical cancer in women and prostate cancer in men, respectively [6,10,11]. Significantly, there is an increased risk for HIV seroconversion in both women and men following infection with *T. vaginalis *[12-17].

On the other hand, *T. tenax *is a commensal of the human oral cavity found under conditions of poor oral hygiene and advanced periodontal disease. Its prevalence in the mouth ranges from 4% to 53% [18]. Interestingly, both *T. vaginalis *and *T. tenax *have recently been reported to be associated with broncho-pulmonary infections in patients with *Pneumocystis carinii *or with underlying cancers or other lung diseases [18-24]. Although speculative to date, the organisms of both species are believed to enter the respiratory tract by aspiration from the oropharynx. While lung infection by the oral trichomonads can be envisioned, the mechanisms by which the urogenital parasites establish residence in the oral cavity for subsequent oropharyngeal and respiratory infections is unclear. Furthermore and importantly, these reports question the extent of the genetic interrelatedness and host site tropisms between these two species.

The phylogenetic analyses based on the rRNA and class II fumerase gene sequences have shown that *Trichomonas *species formed a closely related clade, including isolates of *Trichomonas gallinae*, *T. tenax*, and *T. vaginalis *[25,26]. Given the common host specificity of *T. vaginalis *and *T. tenax*, and the relatedness with respect to rRNA sequences, we felt it important to attempt to determine the extent of genetic identity between the two species. One strategy by us was to identify uniquely-expressed genes of *T. vaginalis *that may represent determinants that contribute to urogenital virulence and pathogenesis. We, therefore, used two approaches. The first involved the subtraction cDNA library approach and the second involved screening a cDNA expression library with pooled patient sera adsorbed with *T. tenax *antigens. We hypothesized that *T. vaginalis *and *T. tenax *would be significantly genetically unrelated to permit isolation of many uniquely-expressed genes of *T. vaginalis*. However, to our surprise, while a few *T. vaginalis *genes were identified, the genes were found to be identical with those of *T. tenax*. We determined that the isolated *T. vaginalis *genes had increased amounts of mRNAs, indicating elevated expression at the transcriptional level. While functional analyses of these up-regulated genes may provide insight about the role of these proteins in trichomonal virulence, our data suggest that both *T. vaginalis *and *T. tenax *have remarkable genetic identity but different rates of gene expression.

## Results

### PCR-based cDNA subtractive hybridization

We have successfully used the PCR-based cDNA subtraction method to isolate differentially expressed cDNAs among two different cDNA populations called tester (*T. vaginalis*) and driver (*T. tenax*) [27]. The driver cDNA population is subtracted from the tester cDNA population by hybridization, and the cDNAs present only in the tester population are enriched and amplified by PCR. This one-way subtraction approach was used to enrich for *T. vaginalis *genes that were absent in *T. tenax*. One drawback with this method is the bias in subtraction based on the transcript levels present in the two cDNA populations being compared. In these experiments we found a high efficiency of subtraction as evidenced by the β-tubulin gene amplification from subtracted and unsubtracted cDNA populations (data not shown). After subtractive hybridization, several cDNAs that were up-regulated in *T. vaginalis *were identified by dot-blot analysis. Cloning and subsequent sequencing of the numerous rescued cDNAs revealed that thirty of the clones were independent, perhaps indicative of efficient subtractive hybridization. A BLAST search revealed that the nucleotide sequences of 14 specific clones were completely identical to the known *T. vaginalis *genes (Table 1), and some of the clones were duplicates. In one case a clone was found in triplicate. The up-regulated genes exhibited homologies with the genomic sequences or expressed sequence tags encoding various functional classes of proteins. The adhesin AP65 (decarboxylating malic enzyme) [28], numerous other metabolic enzymes, and genes involved in cytoskeletal rearrangements were among the apparent uniquely-expressed genes. Interestingly, three genes of the GAPDH multigene family were recovered.

**Table 1:** Genes from subtraction libraries

genome ID		protein	property/function
1.	83711.m00144	Profilin A related	cytoskeletal rearrangement
2.	97241.m00125	Malic enzyme (cytosol)	metabolism
3.	82114.m00023	Actin-related protein	cytoskeletal rearrangement
4.	87955.m00248	Alcohol dehydrogenase 1	metabolism
5.	96423.m00213	lectin repeat family protein	unknown
6.	88613.m00095	TvP14 (fibronectin-like protein-1)	unknown
7.	85938.m00080	CDC42 homolog	surface cell division cycle -GTP-binding protein
8.	85736.m00011	Profilin A related	cytoskeletal rearrangement
9.	83363.m00072	CP3, cysteine protease 3	unknown
10.	92775.m00058	fructose bis-phosphate aldolase	metabolism
11	92066.m00127	AP65-1	adhesin protein
12.	92321.m00066	GAPDH	metabolism
13.	135865.m0003	GAPDH	metabolism
14.	94493.m00018	GAPDH	metabolism
15.	110112.m00002	hypothetical protein 2	unknown
16.	80829.m00126	hypothetical protein	unknown

In the second approach, triplicate screens with adsorbed pooled patient sera of a cDNA expression library revealed thirteen cDNAs, which gave only 7 total genes, again including GAPDH (Table 2). Of particular interest was that GAPDH and hypothetical protein 2 were both found to be identical to those from the subtraction library above (Table 1).

**Table 2:** Genes from screening cDNA library with adsorbed patient sera

Clone number and ID		Protein	property/function
1.	N19, N29	GAPDH^1^	metabolism
2.	13, 25, N3	hypothetical-2^1^	unknown
3.	16, 23, 331	hypothetical-3	unknown
4.	27, 29	hypothetical-4	unknown
5.	33	phosphoglycerate kinase	metabolism
6.	10	aminotransferase	metabolism
7.	I40	f-actin capping protein	actin-cytoskeletal rearrangements

^1 ^GADH and hypothetical protein 2 are same as subtraction and secretome. GAPDH and hypothetical protein 2 are upregulated in expression upon parasite contact with VECs.

### RT-PCR confirms increased gene expression

**Figure 1:**
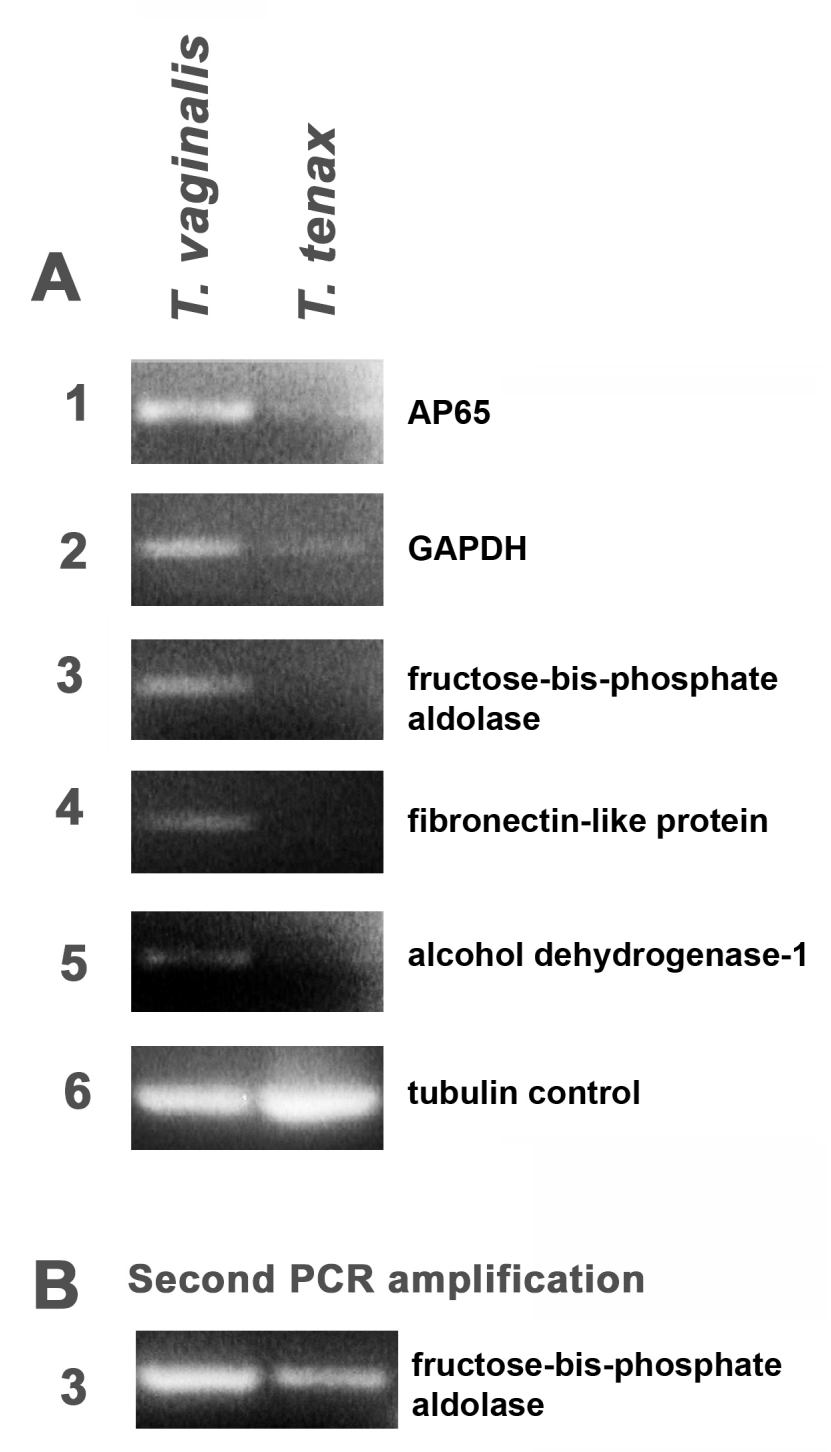
**Confirmation of gene expression patterns in *T. vaginalis *and *T. tenax *by semi-quantitative RT-PCR analyses**. Total RNA from *T. vaginalis *and *T. tenax *was isolated using Trizol reagent and RT-PCR was performed using gene-specific primers. Part A shows the PCR product after 22 cycles, separated on 1% agarose ethidium bromide gel. Part B depicts the re-amplified PCR product for fructose-bis-phosphate aldolase.

**Figure 2:**
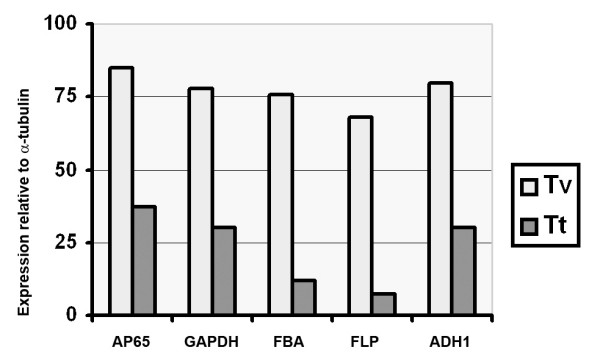
**The gene expression pattern relative to α-tubulin gene as a housekeeping control**. The bar graph shows the relative amounts of RT-PCR products for the five select genes. The values were obtained by scanning the bands from pictures of agarose/ethidium bromide gels using Scion Image beta program. FBA-fructose-bis-phosphate aldolase, FLP- fibronectin-like protein, ADH-alcohol dehydrogenase 1.

Figure 1A shows relative levels of transcription of representative genes that were analyzed by semi-quantitative RT-PCR. The PCR products were separated and visualized on ethidium bromide (EtBr)-stained gels. Intensities and amounts of bands of the PCR products were absent for fructose-bis-phosphate aldolase, fibronectin-like protein, and alcohol dehydrogenase (numbered 3 through 5) or considerably decreased as for AP65 (decarboxylating malic enzyme) and GAPDH (numbered 1 and 2) in *T. tenax *parasites when compared with RT-PCR products derived from *T. vaginalis *handled identically. Given the presence of decreased amounts of transcript for AP65 and GAPDH, we wanted to examine whether the other genes without visible EtBr-stained bands would be detected through a second round of PCR amplification. Figure 1B presents PCR results for fructose-bis-phosphate aldolase with increased amounts of transcript. Similar results were obtained for the fibronectin-like protein and alcohol dehydrogenase 1. Scion image scans of each of the genes through a second round of PCR for each of the genes is presented in Figure 2 and shows the elevated expression for these genes relative to a-tubulin. Compared to *T. tenax *RT-PCR products, the range of increased expression varied from approximately two-fold for AP65 to nine-fold for the fibronectin-like protein-1. These data reaffirm the up-regulation of genes identified by the subtraction library. Next, a partial sequence was amplified for each of the genes analyzed by RT-PCR in *T. tenax*, and the sequence data revealed that the *T. tenax *genes were identical in sequence with that the *T. vaginalis *genes. Collectively, these data indicate that there is high sequence identity between *T. vaginalis *and *T. tenax *and that a distinguishing feature between these two species is the elevated levels of gene transcription by *T. vaginalis*.

## Discussion

In this study we used the approach of suppression subtractive hybridization technique to attempt to identify uniquely-expressed genes of *T. vaginalis *that may represent determinants that contribute to urogenital virulence and pathogenesis. In addition, we also used a second approach and screened a cDNA expression library with pooled patient sera adsorbed with *T. tenax *antigens to identify the uniquely-expressed genes of *T. vaginalis*. Given the fact that *T. tenax *is usually regarded as a harmless commensal of the human mouth, and *T. vaginalis *and *T. tenax *have the same host specificity but different colonization sites [30], we expected to identify many *T. vaginalis *uniquely-expressed genes through our approaches. To our surprise and contrary to our hypothesis, we identified no genes that were unique to *T. vaginalis*. Indeed, the very few genes that were obtained by both approaches were then found to be present in *T. tenax*, but the genes were increased in expression in *T. vaginalis *(Tables 1 and 2).

Confirmation of the expression of select genes using semi-quantitative RT-PCR revealed that all the genes that were identified by the *T. vaginalis *subtraction library and cDNA library with adsorbed patient sera were also present in *T. tenax*, albeit at lower rates of expression. An earlier study involving the characterization of two-dimensional immuno-electrophoretic patterns of different trichomonad species also showed high similarities between *T. vaginalis *and *T. tenax *[31]. Of the 5 transcripts whose relative abundance was found to vary significantly, the AP65, GAPDH, and hypothetical protein 2 were recently found to be secreted or released during growth of *T. vaginalis *[29]. Equally noteworthy is that these proteins are upregulated in expression upon parasite contact with vaginal epithelial cells [32]. The up-regulated expression in *T. vaginalis *of proteins by various environmental cues, such as adherence, may suggest an important role as virulence factors in urogenital infection. Indeed, AP65 is a prominent adhesin of *T. vaginalis *important for attachment to vaginal epithelial cells [33-35].

While we expected a high genetic divergence between the oral and urogenital trichomonads, the high genetic identity between *T. vaginalis *and *T. tenax *was surprising. While whole genome comparisons are needed, these are not currently available. It is reasonable to hypothesize, therefore, that if in fact these species are highly related or even identical, the minor variance between the two may have resulted due to their introduction and residence in distinct environmental niches. Contributing to their survival in different mucosal sites may be the important distinguishing feature of higher rates of transcription by *T. vaginalis *compared to *T. tenax*, which may have resulted from the environments imposing unique survival pressures. Alternatively, the increased expression levels may result from the gene duplication in the pathogenic species. In fact, the genome sequencing project has revealed that *T. vaginalis *genome has undergone expansion on a scale unprecedented in unicellular eukaryotes [36], and such gene family expansions are likely to improve the specific adaptation of the organism to its environment [37]. Furthermore, there are variations between the 5S rRNA genes of *T. vaginalis *and *T. tenax *(personal communication). This fact may explain the expression levels of identical genes within the two highly related species. Without a doubt, such a modification in the gene inventory in the genomes of pathogens would be an important evolutionary signal. In fact, several studies have shown a relationship between virulence, differential gene acquisition and copy number, and gene expression in both bacteria and viruses [38], and this may be what resulted to distinguish *T. vaginalis *from the oral trichomonad. Therefore, it is altogether reasonable that the levels of transcription and synthesis of proteins in these two trichomonad species may account for adaptability for survival in their respective oral cavity and urogenital regions.

Finally, our results may begin to delineate recent findings regarding how both *T. vaginalis *and *T. tenax *are associated with broncho-pulmonary infections in patients with *Pneumocystis carinii *or with underlying cancers or other lung diseases [18-24]. As mentioned above, the respiratory-lung environment is itself distinct from the oral cavity and urogenital region, but this niche obviously permits survival of both regardless of the extent of gene expression for *T. vaginalis *and *T. tenax*. While lung infection by the oral trichomonads can be envisioned, the mechanisms by which the urogenital parasites establish residence in the oral cavity for subsequent oropharyngeal and respiratory infections is unclear. Future considerations must now be given regarding methods of transmission of *T. vaginalis *into lung tissues. It is possible that this parasite colonizes the oral cavity through oral sex and survives for extended periods prior to aspiration and infection. It is equally theoretically possible that *T. tenax *is a genetic variant of *T. vaginalis *distinguished by rates of gene transcription. It may be unlikely that *T. tenax *infects the urogenital region of women. One reason for this may be that this trichomonad is nonadherent to HeLa epithelial 9 cells [39] and vaginal epithelial cells (not shown). As *T. tenax *has the genes encoding adhesins, such as AP65 [32-35], this inability to bind epithelial cells, a property preparatory to infection and colonization, may help explain the tropism of *T. tenax *to the oral cavity. It is conceivable that the decreased level of expression of these adhesin genes in *T. tenax *accounts for this inability to adhere to vaginal epithelial cells. These possibilities will require future experimental examination. We feel that this report now questions the extent of the divergence between these two species and shows high genetic identity between *T. vaginalis *and *T. tenax*.

## Conclusion

Using two approaches did not yield any *T. vaginalis *unique genes, suggesting strongly there is a high genetic identity between *T. vaginalis *and *T. tenax*. For all of the genes originally identified and examined as unique to *T. vaginalis*, the genes were found to be identical in *T. tenax*. We found higher rates of transcription in *T. vaginalis *compared with *T. tenax*. Our data may help explain recent reports on the respiratory infections by both of these trichomonal species. Finally, attention needs to be given to the possibility that *T. tenax *is a genetic variant of *T. vaginalis*.

## Methods

### Parasites

The fresh clinical isolates of *T. vaginalis *UT00-40 and T016 were grown in batch culture at 37°C no more than three weeks in trypticase-yeast extract-maltose (TYM) medium supplemented with 10% heat-inactivated horse serum [40]. The isolate T016 was used for construction of the expression cDNA library that was used for screening with *T. tenax*-adsorbed pooled patient sera, as described below. The *T. tenax *Hs-4:NIH was grown in LYI Entamoeba medium supplemented with 10% heat-inactivated fetal bovine serum as recommended by ATCC. The *T. tenax *isolate was confirmed using the PT3 sense primer (5'-AGTTCCATCGATGCCATTC-3') and the PT7 antisense primer (5'-GCATCTAAGGACTTAGACG-3') [41].

### PCR-based cDNA subtractive hybridization

Total RNA was extracted from *T. vaginalis *UT00-40 and *T. tenax *organisms using Trizol (Invitrogen, Carlsbad, CA). The double-stranded cDNAs were synthesized from 1 μg total RNA of each group using a Smart PCR cDNA synthesis kit (BD Clontech, Mountain View, CA) and were used for suppression PCR-based cDNA subtractive hybridization using a PCR-select cDNA subtraction kit (BD Clontech). The cDNAs prepared from *T. tenax *and *T. vaginalis *were regarded as driver and tester, respectively, and the driver cDNA population was subtracted from the tester cDNA population. Suppression PCR was performed to prepare the cDNA pool, enriched for genes accumulated in *T. vaginalis *(forward-subtracted). The resultant tester-specific cDNAs were amplified by PCR, and cloned into pGEM-T-easy vector (Promega Corp., Madison, WI). The detailed procedures were described in the protocol of the PCR-select cDNA subtraction kit (BD Clontech). The subtracted cDNA fraction was cloned into a TA vector and transformed into *Escherichia coli *to create an enriched *T. vaginalis *cDNA library.

### Sequencing and analysis

Colonies were randomly selected, and plasmids were prepared using a Miniprep kit (QIAGEN, Valencia, CA). The cDNA inserts were verified by restriction digestion, and the clones were sequenced in the Washington State University institutional DNA-sequencing facility. Sequence data was compared with the GenBank database using a BLAST program.

### RT-PCR analysis of selected genes

Differential expression of a subset of cloned genes was confirmed by semi-quantitative RT-PCR. Total RNA from *T. vaginalis *and *T. tenax *parasites was reverse transcribed with the oligo(dT)_15 _primer using Superscript II reverse transcriptase (Invitrogen), according to the manufacturer's protocol. PCR amplification of cDNA was carried out using gene-specific primers. The trichomonad a-tubulin gene was used as an internal control. Twenty-two cycles were used for amplification of specific genes. As there was no clear band detected for fructose-bis-phosphate aldolase gene, the initial PCR product was used as a template to re-amplify the product if any, for 30 cycles. All RNA samples without reverse transcription were also used for PCR to detect genomic DNA contamination, and at no time was DNA detected. PCR products were visualized on EtBr-stained agarose gels. The band intensity was quantitated using the Scion image beta program. The PCRs were carried out at four different times to verify the reproducibility of results. The result from a representative experiment is used here.

### Screening of the cDNA library using pre-adsorbed *T. vaginalis *patient serum

Specific, adsorbed anti-*T. vaginalis *patient antibodies were obtained by incubation of the pooled patient sera with immobilized nitrocellulose membranes first treated with a preparation of total *T. tenax *proteins. Briefly, 1 × 10^9 ^washed *T. tenax *parasites in PBS were lysed by sonication and boiled in 2 ml of electrophoresis sample buffer. The nitrocellulose was then saturated with lysate for 3 h followed by washing with PBS. Lysate was used with other membranes until depletion of the proteins was visibly detected after SDS-PAGE and staining of gels. The membranes were then saturated with blocking solution (PBS containing 0.05% Tween-20 and 10% skim milk) for 1 h. Membranes were then incubated 2 h with 100 ml of pooled patient sera diluted 1:50 in PBS containing 0.05% NaN_3 _and 5% skim milk. The adsorbed sera was removed, and bound antibodies were eluted by 3 washes of membranes in 100 ml of PBS-0.1 M glycine, pH, 3.0. The adsorbed diluted patient sera were treated 3 separate times. The *T. vaginalis *patient antibodies solution was used to screen a previously-obtained λZAP II *T. vaginalis *cDNA library. Fusion proteins were induced with isopropyl-β-D-thiogalactopyranoside (IPTG) and recombinant plaques detected with adsorbed antibodies. After cloning and purification of reactive plaques, the corresponding pBluescript plasmids were excised. The recombinant plasmids were transformed into *E. coli *XL-1-Blue. Plasmids containing the cDNA coding for the *T. vaginalis *reactive recombinant proteins were sequenced.

## Abbreviations

ADH: alcohol dehydrogenase 1; AP65: adhesin protein 65-kDA; GAPDH: glyceraldehydes-3-phosphate dehydrogenase; FBA: fructose bis-phosphate aldolase; FLP: fibronectin-like protein; PCR: polymerase chain reaction; STD: sexually transmitted disease.

## Authors' contributions

AK and VM performed the subtraction, differential expression, and sequencing data. All authors contributed to the writing of this manuscript. JFA contributed to the design of the experiments and offered suggestions during the experiments. All authors read and approved the final manuscript.
